# Integrating Virtual Reality, Neurofeedback, and Cognitive Behavioral Therapy for Auditory Verbal Hallucinations (Hybrid): Protocol of a Pilot, Unblinded, Single-Arm Interventional Study

**DOI:** 10.2196/63405

**Published:** 2025-04-01

**Authors:** Jessica Spark, Elise Rowe, Mario Alvarez-Jimenez, Imogen Bell, Linda Byrne, Ilvana Dzafic, Carli Ellinghaus, Suzie Lavoie, Jarrad Lum, Brooke McLean, Neil Thomas, Andrew Thompson, Greg Wadley, Thomas Whitford, Stephen Wood, Hok Pan Yuen, Barnaby Nelson

**Affiliations:** 1 Orygen Parkville Australia; 2 Centre for Youth Mental Health University of Melbourne Parkville Australia; 3 School of Psychology Deakin University Burwood Australia; 4 The Cairnmillar Institute Hawthorn East Australia; 5 Centre for Mental Health and Brain Sciences Swinburne University of Technology Hawthorn Australia; 6 School of Computing and Information Systems University of Melbourne Parkville Australia; 7 School of Psychology University of New South Wales Sydney Australia; 8 School of Psychology University of Birmingham Edgbaston United Kingdom

**Keywords:** psychosis, first episode psychosis, schizophrenia, virtual reality, neurofeedback, EEG, auditory verbal hallucinations, voices, cognitive behavior therapy, youth mental health, pilot study, paracusias, paracusis, treatment, medication, psychotic disorder, efficacy, neuroscience, psychology, hybrid, adolescent, Australia

## Abstract

**Background:**

Current treatments for schizophrenia and other psychotic disorders have limited efficacy, with high rates of nonresponse to “gold standard” treatments. New approaches are therefore urgently required.

**Objective:**

The aims of this pilot study are to investigate the feasibility, acceptability, safety, and usability of Hybrid treatment (primary aim); and to explore Hybrid’s treatment efficacy and engagement of treatment targets (secondary aim). The primary aim will be assessed via face-to-face user experience surveys on a (self-assessed) 5-point Likert scale (and qualitative open-ended questions) examining: (1) acceptability, (2) helpfulness, (3) engagement, and (4) perceived safety. We will also examine consent and completion rates, and the number of sessions attended. Our threshold for moving on to efficacy trials will be at least 70% of our participants to rate 3 and above (which corresponds to agree or strongly agree) that the intervention package was acceptable, feasible, and safe. The secondary aims will be assessed by observing whether individuals achieve self-directed modulation of high-β neurophysiological activity (neural target) and progression upwards through the VR-based exposure hierarchy (psychological target), and by assessing symptom change scores. This study developed a new treatment approach for auditory verbal hallucinations, a major symptom of psychotic disorders, that integrates advances in psychological therapy (cognitive behavioral therapy for psychosis), technology (virtual reality, VR), and neuroscience (electroencephalography-based neurofeedback).

**Methods:**

Hybrid takes a “symptom capture” approach using individually tailored VR-based exposure exercises. Participants (N=10) will receive the intervention package weekly over 12 face-to-face sessions. Here, participants will be progressively exposed to symptom triggers and develop methods of downregulating neural activity associated with these symptoms (neurofeedback component) while concurrently receiving clinician-delivered cognitive behavioral therapy for psychosis.

**Results:**

As of February 2025, Hybrid has commenced (unblinded) recruitment activities from Orygen clinical services in Northwestern Melbourne, Australia. A total of 75 individuals have been approached and 64 individuals have been prescreened (41 individuals were deemed eligible, 15 individuals were ineligible, and 8 individuals declined or did not respond to contact attempts) and 5 individuals have been included in the study. Of the 5 individuals who have commenced the Hybrid treatment, 4 are actively engaged in the program and 1 individual has withdrawn. We expect recruitment to conclude in July 2025 and for the results to be published in 2026.

**Conclusions:**

The Hybrid study is piloting a novel approach that has the potential to address the shortcomings of current treatments for psychotic symptoms. If there is favorable evidence for the acceptability, feasibility, safety and usability of Hybrid, the study team will move on to efficacy trials.

**Trial Registration:**

Australian New Zealand Clinical Trials Registry ACTRN12624000357550; https://tinyurl.com/24ey8hpy

**International Registered Report Identifier (IRRID):**

PRR1-10.2196/63405

## Introduction

### Overview

Psychosis is the distinguishing feature of schizophrenia spectrum disorders and a frequent manifestation of mood and substance use disorders [1]. It is characterized by alterations in thoughts and perceptions, often taking the form of positive symptoms such as delusions, hallucinations [2], and disorganized thinking [3], as well as negative symptoms such as blunted affect, poverty of speech, and withdrawal from social and occupational activities [4]. First episode psychosis (FEP) onset most commonly occurs in late adolescence and early adulthood [5], with auditory verbal hallucinations (AVHs) being the most prevalent type of positive symptom reported in this clinical population (≈80%) [6,7]. AVHs are typically characterized by hearing voices that are perceived as distinct from the person’s own thoughts. Such experiences are often a cause of significant distress and disruption to daily functioning [8], making AVHs an important target for treatment. Furthermore, evidence supports the benefits of early intervention following the first onset of psychotic symptoms with the duration of untreated psychosis (ie, the time between first psychotic symptoms to antipsychotic treatment) consistently predicting poorer long-term outcomes in longitudinal studies [9,10].

### Current Treatments for Psychosis

Current treatment recommendations for psychosis include antipsychotic medications, psychosocial support, and psychological treatment, specifically, cognitive behavioral therapy for psychosis (CBTp) [11-13]. Unfortunately, all currently available treatments are only moderately effective. First, the efficacy of antipsychotic medication for the treatment of positive symptoms of psychosis is not optimal. For example, while commonly prescribed antipsychotics can be significantly more effective than placebo, these medications are accompanied by side effects such as weight gain, sedation, and extrapyramidal symptoms which lead to high incidences of patient nonadherence [2]. A 2019 meta-analysis including 16 randomized controlled trials of antipsychotics and >6200 patients with schizophrenia found that 38% (n=2364) of patients met the criteria for nonresponse and two-thirds showed only “minimal improvement” [14]. Second, the effect sizes for psychological treatments, such as CBTp, tend to be small (≈0.4) and limited in long-term efficacy [2]. Both pharmacological and psychological interventions have higher rates of nonresponse [14] and disengagement [15] in patients with earlier onset of illness highlighting the importance of targeting FEP populations and developing effective and reliable treatments for early intervention. New treatment development, both pharmacological and psychological, is therefore urgently required.

### “Cold Cognitions”

One of the limitations of psychological therapies such as CBTp may be that they tend to rely on abstract self-reflection (or “cold cognitions”) which can be detached from the actual in vivo experience of symptoms (or “hot cognitions”) [16]. This limitation may be particularly relevant for young people in the early stages of psychotic disorder, many of whom find it challenging to engage in this form of self-reflective therapy [17]. Borrowing from the “symptom capture” approach used in other mental disorders, the effectiveness of CBTp may be improved if it is applied while the symptom is actively occurring [18] and collaboratively addressing the symptom that has been activated. The benefit of actively eliciting “hot” cognitions has been demonstrated in the treatment of anxiety disorders and obsessive-compulsive disorder (OCD) [19]. In these disorders, “exposure and response prevention” (ERP) is a key feature of gold-standard psychological treatment [20]. This therapeutic approach involves actively eliciting a degree of anxiety by exposing the patient to symptom triggers and addressing the cognitive and behavioral reactions together with the therapist. Once a degree of mastery and tolerance has been achieved over a low-level, anxiety-inducing trigger, the patient and therapist collaboratively proceed to higher levels of an exposure hierarchy.

### Virtual Reality

One advance in technology that may help anchor treatment to the in vivo experience of symptoms is virtual reality (VR). VR is an interactive computer-generated experience in which the user wears a headset that immerses them in a simulated environment. The VR system is able to create sensory illusions that mimic reality and elicit brain and behavioral responses that parallel those that occur in the real world [21]. VR environments have been found to be useful in the treatment of mental disorders such as social anxiety (for review see Chard and van Zalk [22]), phobias (for review see Kothgassner et al [23]), posttraumatic stress disorder (PTSD; for review see Maples-Keller et al [24]), and as a treatment of psychotic symptoms such as paranoia [25] (see Valmaggia et al [26] for review). Similar to cognitive behavioral therapy (CBT) for anxiety and OCD, this therapeutic technique is founded on exposure principles, where a person is presented with feared, anxiety-provoking, or symptom-inducing stimuli in a graded manner within a controlled environment to enable them to gradually develop strategies to cope when triggers arise in the course of daily life. VR studies for PTSD have found that immersion (ie, a subjective sense of presence in the VR environment) and personalization of the VR environment (ie, tailoring the VR environment to the person’s specific and idiosyncratic triggers) may lead to better outcomes, as therapy is more likely to generalize to real-world situations [27]. Indeed, a meta-analysis of VR-assisted exposure therapy for anxiety disorders showed a large overall effect size for VR exposure therapy compared with (mostly waitlist) control conditions (Cohen *d*=1.11, SE 0.15, 95% CI 0.82-1.39) [28]. Furthermore, research on the use of VR for psychosis treatment has indicated the approach can be feasible, acceptable, and effective for improving some symptoms including positive symptoms such as paranoia and hallucinations [29].

For the specific treatment of AVHs, the small number of VR trials to date have all involved avatar therapy, a related approach that involves the creation of a computerized, audio-visual avatar that mimics the hallucinated voices normally experienced by participants, either on a computer screen [30] or using a head-mounted display [31,32]. During these sessions, the participant is supported by the therapist to engage in a dialogue with the created personalized avatar and to develop strategies to respond to the voices. At the same time, the avatar voice and expressions are gradually adjusted from their normal content/tone (eg, abusive) to be friendly and supportive, controlled by the therapist [33]. A 2020 meta-analysis showed favorable results for avatar therapy in patients with schizophrenia compared with supportive counseling on measures of mental state, level of insight, and quality of life [34]. However, while avatar therapy involves artificially recreating an analog AVH experience, the use of the VR environment to elicit symptoms naturally (as is used in traditional ERP treatments) has not yet been explored.

### Neurofeedback

Another technological advance, neurofeedback, has proven useful in the treatment of a number of disorders and has shown promising results for psychotic symptoms [35,36]. Neurofeedback is a technique in which individuals use a brain-computer interface to control their own brain activity by receiving real-time feedback on their neural activity. Neurofeedback has most commonly been provided via electroencephalography (EEG) or real-time functional magnetic resonance imaging (rtfMRI). In an example of EEG neurofeedback, individuals receive real-time information on their brain activity, often presented as a bar graph, where the height of the bar represents the power of oscillatory activity in a given frequency band (eg, α or β) measured in terms of power (ie, amplitude squared). This visual display allows participants to try to gain control over their brain activity by manipulating the visual feedback. Participants are typically provided with directions or mental techniques to control their brain activity, are encouraged to apply and adapt their own strategies, and to use the visual feedback display as a guide [37].

The ability of healthy individuals to manipulate their neural activity using neurofeedback has repeatedly been demonstrated [38-40] and is suggested to have positive impacts on cognitive and emotional domains, including the improvement of working memory [40], intelligence [38], and identification of emotional prosody [41]. This has led to neurofeedback’s clinical application to a range of psychiatric or neurological disorders, with meta-analyses reporting benefits for attention deficit hyperactivity disorder [42], depression [43], PTSD [44], subclinical OCD [45], and epilepsy [46]. The findings in patients with medication-resistant epilepsy are particularly striking, with neurofeedback demonstrating efficacy in averting seizures once patients learn to anticipate their onset, and with a transfer effect evident even years later [42,46].

The use of neurofeedback in psychotic disorders is increasing in popularity, with a small number of studies published to date (for review see [36]). All studies have found that patients with schizophrenia were able to self-regulate their neural activity, although evidence for a transfer effect (that is, the ability of participants to employ the strategies they have learned during neurofeedback to control their neural activity in the absence of neurofeedback) has yet to be clearly demonstrated. A particularly promising study using rtfMRI indicated a transfer effect for patients with schizophrenia with treatment-refractory AVH after a 2-week training period [47]. Interestingly, the posttraining increase in functional connectivity between brain regions associated with AVH (the left superior temporal gyrus and inferior prefrontal gyrus) was associated with a reduction in AVH symptoms over the training period. As Nan et al [48], recently concluded, “these positive outcomes suggest that such intensive neurofeedback training may provide new insight into the treatment of schizophrenia and thus deserves further study to fully examine its scope.”

While rtfMRI-based neurofeedback (like that used in the aforementioned study) can be costly and uncomfortable (due to the space restrictions), EEG-based neurofeedback offers the benefits of portability, cost-effectiveness, and higher patient acceptability. EEG has the further advantage of capturing oscillatory dynamics associated with neural functioning, which cannot be detected using functional MRI owing to the slow temporal latency of the hemodynamic response [49,50]. For patients with FEP, studies on EEG frequency bands (α: 8 to 12 Hz; β: 13 to 30 Hz; θ: 4 to 7 Hz; δ: 0.5 to 4 Hz; low γ: 30 to 50 Hz; and high γ: 50 to 90 Hz) have reported several abnormalities across all 6 bands [51]. Oscillations in the high β band range (approximately 18-30 Hz) are associated with autonomic nervous system hyperarousal and anxiety, which are recognized as one of the most common subjective features of AVH [52,53]. Previous studies have found that reducing high β band activity (and hyperarousal) is associated with a decrease in psychotic symptoms in patients with schizophrenia, including AVH [48,54,55]. Thus, the use of EEG-based neurofeedback targeting high β band activity represents an important target for the treatment of AVH.

### The Hybrid Study

This study, Hybrid, will investigate the potential of a novel treatment approach to AVH in young people with FEP that, if successful, could be incorporated into an individual’s broader health care program. Our integrative model combines psychological therapy (CBTp), new immersive technologies (VR), and neuroscience (neurofeedback) in an individualized symptom capture (or ERP) treatment approach. The term “Hybrid” is used to reflect the integrated approach of treatment where each element has been selected to strengthen the effectiveness of the treatment regime as a whole.

A pilot study is required to investigate the following aims: (1) to determine whether Hybrid is feasible, acceptable, and safe; (2) to examine participants’ experience of the intervention package and whether it can be modified to increase probability of clinical utility; (3) to explore if there is a signal of clinical benefit in response to Hybrid treatment; and (4) to examine whether Hybrid engages specific neural (modulating high β band activity) and psychological (moving up a symptom-eliciting exposure hierarchy) targets (proof of concept). Hybrid will be an unblinded, single-arm intervention study.

## Methods

### Participants

Ten participants with a diagnosed FEP will be recruited via telephone from Orygen’s Early Psychosis Prevention and Intervention Centre, headspace youth mental health services, and the Orygen Clinical Trials Unit database which serve the Northern and Western suburbs of Melbourne, Australia. Help-seeking participants with FEP from the community will also be recruited. Recruitment will involve phone calls, email, and SMS contact with potential participants. Participants will receive a manually initiated reminder SMS or phone call the day before participation sent by the study clinician or research assistant.

### Inclusion Criteria

The inclusion criteria are as follows:

- Aged 15-26 years inclusive.
- Sufficient fluency in English to engage in psychological therapy with an English-speaking therapist, and to understand the study assessments.
- Ability to give informed consent and adhere to study procedures. Parental or guardian consent will be obtained for participants younger than 18 years.
- Psychotic threshold AVH in lifetime as measured by the Comprehensive Assessment of At Risk Mental States (5 or 6 on the severity and ≥4 on frequency for longer than 1 week [56]).
- A rating in the past year of ≥1 on the Psychotic Symptom Rating Scales hallucinations scale [57], corresponding to voices occurring at least once/week. The purpose of this is to ensure that participants with reasonably frequent hallucinations are included in the study, as the experience of symptoms during Hybrid sessions will optimize the study’s test of outcomes of interest.

### Exclusion Criteria

The exclusion criteria are as follows:

- Documented history of head injury, seizures, or other significant neurological illness.
- Documented history of intellectual disability.
- Visual impairment precluding the ability to view VR scenarios.

### Ethical Considerations

Ethics approval for Hybrid has been obtained from The Royal Melbourne Health Human Research Ethics Committee (number 2022.116). This study has ethics approval via the Melbourne Health Human Research Ethics Committee (HREC/76936/MH-2022) for research involving humans and access to patient medical records and information. Participants (or their caregiver/guardian if younger than 18 years) must provide informed consent to participate in the Hybrid treatment. Participants are free to withdraw at any time, with the knowledge that doing so has no impact on their routine treatment. All data from the study is deidentified and only the study investigators have access to this information. This means that each participant’s data will have a unique code which only the study team members will have access to. Each participant will be reimbursed AUD $50 (US $31.72) per research interview visit as well as AUD $20 (US $12.69) per EEG recording session.

### Data Management and Imputation Strategy

To address attrition or missing values, we will code these as “N/A.” The current follow-up protocol is designed to maximize data collection; however, if the missing data are substantial, we will apply multiple imputation methods.

### Key Components of the Hybrid Treatment Model

#### Psychological Therapy

The CBTp approach used in Hybrid relies on hierarchical exposure principles in order to trigger cognitive and affective reactions that occur while experiencing symptoms, that is, “hot cognitions” during symptom activation [58]. As outlined above, this approach may maximize the impact of psychological therapy, as has been observed in other disorders.

#### Virtual Reality

The VR paradigm that will be used to elicit AVH is inspired by Stinson et al [59] who used a real-world setting comprising computer-generated characters of both sexes and several ethnicities and background sounds simulating the environment. Our VR approach is based on the CBTp framework, beginning with developing a shared understanding (functional analysis) between the participant and therapist of the antecedents of hallucinatory episodes (eg, presence of other people and lack of meaningful activity) and habitual responses to them (eg, talking back to voices, avoidance). The participant and therapist will then select a template scenario from preexisting VR environments (eg, supermarket and bedroom) developed in consultation with a small group of current and previous service users. Various aspects of the VR environment will be modified to personalize it for the antecedents the individual has identified and to create a hierarchy of symptom-provoking situations. These will include such aspects as the number of people present, proximity and gaze intensity of people, context (interpersonal interaction, indoors, outdoors, time of day or night), sounds, and the presence or absence of “safety strategies” to manage symptoms (eg, mobile phone). For each participant, there will be 5 scaled variants of the personalized scene graded on a 0-100 scale of subjective distress (ranging between 0 and 20 [low], 21 and 40 [moderate], 41 and 60 [high], 61 and 80 [very high], and 81 and 100 [severe]). There are a number of benefits of the VR approach. (1) It provides a controlled environment that can be stopped at any moment, which is not always feasible in real-life exposure settings. (2) It can be designed in a manner that is highly personalized to the individual, which may maximize therapeutic potential. (3) It is a treatment approach that may be particularly suited to young people, given its engaging and interactive nature. (4) It is becoming increasingly feasible for translation into clinical services, given the dramatic reduction in the cost of VR equipment and the fact that VR devices are becoming increasingly portable.

#### Neurofeedback

EEG will be used to provide real-time neurofeedback to participants while experiencing AVH.

EEG will be acquired using the Muse Headband 2 attached to the Oculus Rift S VR headset. The Muse Headband 2 acquires EEG via 4 electrodes placed over the prefrontal (forehead) and temporal-parietal areas of the brain (electrodes AF7, AF8, TP9, TP10 in the traditional 10-20 electrode system) and collects data at a sampling rate of 500 Hz (with a fifth reference electrode in the position of FPz). The Muse device has been shown to yield frequency-based components of the EEG signal comparable to the research-grade EEG system Biosemi Active Two with the additional benefit of live-streaming capabilities and neurofeedback integration [60]. In Hybrid, the neural target will be the neurophysiological activity associated with autonomic nervous system hyperarousal, specifically, power in the high β range (18-30 Hz) as discussed above (see below for further details).

### Intervention Design

**Figure 1:**
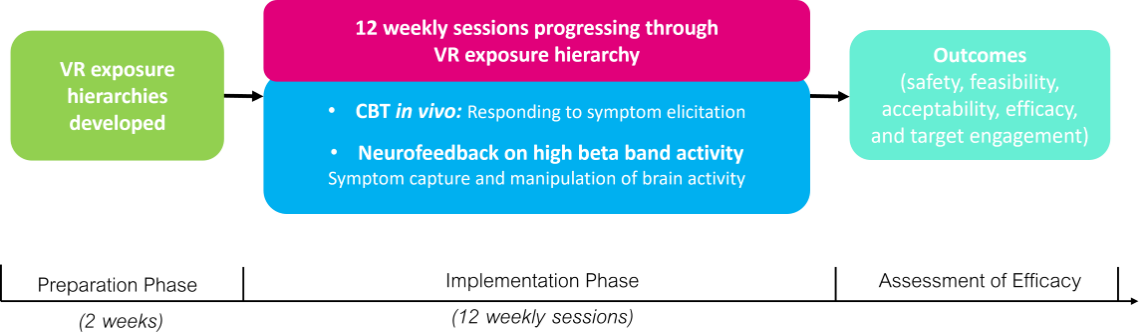
Hybrid design. CBT: cognitive behavioral therapy; VR: virtual reality.

**Figure 2:**
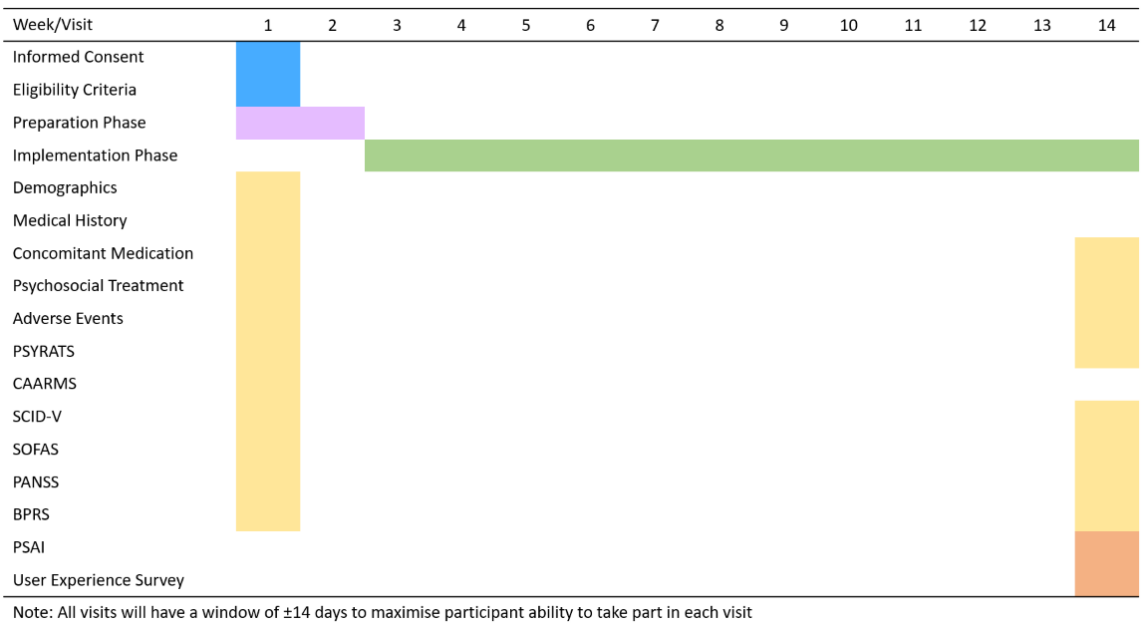
Hybrid schedule of assessments. BPRS: Brief Psychiatric Rating Scale; CAARMS: Comprehensive Assessment of At Risk Mental States; PANSS: Positive and Negative Syndrome Scale; PSAI: Pilot Study Assessment Instrument; PSYRATS: Psychotic Symptom Rating Scales; SCID-V: Structured Clinical Interview for the DSM-V; SOFAS: Social and Occupational Functioning Assessment Scale.

Hybrid consists of a Preparation phase (face-to-face, approximately 2 weeks) followed by an implementation phase (12 weekly face-to-face sessions based on previous VR study designs [31,61-63]). Figure 1 shows a visual representation of the Hybrid intervention design and Figure 2 presents the schedule of assessments.

### Preparation Phase (Weeks 1-2) Development of the VR Exposure Environment

**Figure 3:**
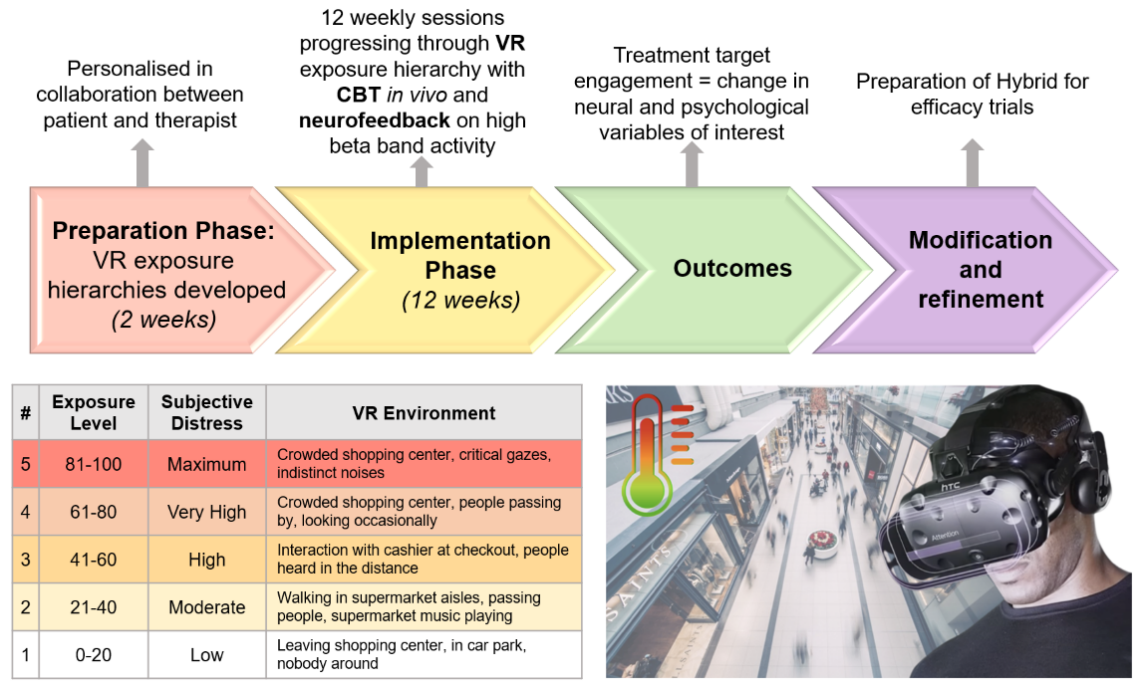
Hybrid VR exposure hierarchy example: Shopping Centre template. Example of VR hierarchy exposure levels (left) based on the shopping center template. The participant is exposed to the exposure environments via the VR headset while concurrently speaking with their therapist (cognitive behavioral therapy for psychosis) and receiving neurofeedback of high β band activity via a visual display of a thermometer (right). VR: virtual reality.

The preparation of the VR exposure hierarchy will be embedded within a CBT framework by using an adaptation of the standard CBT for voices approach [64]. As mentioned above, the participant and therapist will develop a hierarchy of VR environments personalized for the antecedents identified as symptom-provoking. An example of a VR exposure hierarchy is provided in Figure 3. In developing this hierarchy, the participants will rate the degree of subjective immersion (“presence”) they felt in the VR environment using the Immersive Technology Centre-Sense of Presence Inventory [65], a widely used measure in this field. VR environments will only be used if they are rated as inspiring at least “moderate presence,” as this has been found to increase therapeutic effectiveness [27]. Once the VR exposure hierarchies have been developed, participants will commence the implementation phase.

### Implementation Phase (Weeks 3-14)

The implementation phase will consist of weekly sessions delivered over a 12-week period (SD 14 days, Figure 2) and consists of the following two components: (1) CBT in vivo (in response to the VR exposure) and (2) neurofeedback simultaneously presented to the participant via the VR device. The VR apps and sessions can only be accessed by the clinician and administered in person. During these weekly sessions, the therapist will be able to view the VR scenario (via an external monitor) and the neurofeedback thermometer, hear the auditory input, and provide guided assistance to the individual.

### Cognitive Behavioral Therapy in Vivo (in Response to VR Exposure)

The participant will start at the lowest level of their exposure hierarchy and progressively work up the hierarchy over the course of the sessions. The standard approach in ERP interventions will be adopted, which is to achieve a reduction in subjective units of distress to 30/100 before progressing to the next step in the hierarchy. While the participant is exposed to the symptom-eliciting VR scenario, they will engage in CBTp with their therapist (the participant will be wearing “open backed” headphones, allowing them to hear the therapist as well as sounds coming from the VR environment). The CBT will be standard, manualized CBT for voices [64,66,67] dealing with the “hot” cognition reactions elicited by the VR scenario. Key elements include reassurance regarding lack of threat, challenging appraisals of AVH (eg, their perceived power, threat, or dominance; reducing submissive or reactive behaviors), actively responding to AVH, and developing a sense of control over AVH by attempting to delay or reduce their volume or distract from them with a variety of strategies (eg, humming and visualization). Sessions will last 30-45 minutes and will be stopped at any point if requested by the participant.

### Neurofeedback Simultaneously Presented to Participant Via the VR Device

While the participant is being exposed to the VR scenarios and engaging in CBT with a therapist, they will receive visual neurofeedback of electrophysiological activity (power) in the high β band (18-30 Hz) range as measured via EEG. The neurofeedback will be provided to the participant via a single visual image of a thermometer in the corner of the VR scene (ranging from 0 to 100 “degrees”). The temperature of this thermometer is based on the individual’s “baseline” neural activity within the high β band range which is measured at the start of each session prior to entering the VR scenario. The thermometer will increase in “temperature” if high β band power increases. The therapist will inform participants that this thermometer is a visual representation of their current stress levels which will give them a real-time objective indication of how their brains are reacting to the Hybrid therapy. Participants will be instructed to keep their eyes open (so as to remain exposed to the VR scenarios) and to keep the thermometer below the 50-degree reading (ie, “half way”; regulating their neural activity in the desired direction) as an additional motivating goal. In keeping with other neurofeedback studies [18], they will be instructed to devise their own strategy for modulating this neural activity but will be informed that they can discuss different strategies with their therapist. If the thermometer measurement is maintained in the lower half ≥50% of the session time, participants will be made aware of the success of their efforts as positive reinforcement at the end of each of their weekly sessions.

### Integration of Multiple Technologies in One Innovative Treatment Package

Combining the treatment components in Hybrid will engage multiple treatment targets and possible pathogenic mechanisms (psychological, neurophysiological, and neurobiological) simultaneously. There is also likely to be a synergistic effect through the interaction of the individual treatment components that will make the package more than the sum of its parts. To illustrate, the real-time neurofeedback provided to participants and therapists in response to the VR exposure exercises will feed into the content of the CBT therapy, because the participant and therapist will be able to discuss the direct impact the exposure exercises are having in an immediate, non-retrospective fashion. This preserves the benefits of dealing with “hot” cognitive and affective reactions. In turn, the CBT will assist the young person in developing strategies for modulating their neural activity in response to the VR exposure exercises. Moreover, the neurofeedback will dynamically reinforce and boost the effect of CBT via participants being able to see in real time that the CBT is helping change their brain activity. This will create a salient and durable learning experience, contributing to a “real world” transfer effect and motivating the use of CBT skills in the absence of neurofeedback. This tridirectional impact of the treatment components—the way the individual items work together—will amplify their individual contribution to therapeutic gain.

### Measures

#### Primary Outcomes

##### Feasibility and Acceptability of the Hybrid Model

A Pilot Study Assessment Instrument developed by Orygen researchers based on the user experience approach will assess (1) acceptability, (2) helpfulness, (3) engagement, and (4) perceived safety. Items are rated on a self-assessed 5-point Likert scale. Acceptability and feasibility will also be determined by assessing consent and completion rates, the number of sessions attended, the number of dropouts, and completion rates of measures. A high rate of elicited AVH is required to facilitate “hot” cognitions so that the symptoms are targeted directly rather than through abstract self-reflection.

##### Safety of the Hybrid Model

Safety will be assessed by recording adverse events or serious adverse events.

##### Usability of the Hybrid Model

Assessing the experience and usability of the VR environments was conducted using the user experience survey, a specifically adapted 18-item questionnaire from other Orygen VR studies for this study. This measure will be completed at the end of the Hybrid intervention (week 14 of the study). At this point, participants will also complete a semistructured interview about their experience of the intervention and suggestions for modifications to the treatment package.

##### Threshold of the Hybrid Model

Our decision to move on to efficacy trials will be based on the user experience survey, which uses a 5-point Likert scale. We will require at least 70% of our participants to rate 3 and above (which corresponds to agree or strongly agree) that the intervention package was acceptable, feasible, and safe.

#### Secondary Outcomes

##### Auditory Verbal Hallucinations

The hallucinations scale of the Psychotic Symptom Rating Scales will be used to assess changes in AVH severity, frequency, and distress.

##### General Psychopathology

The Brief Psychiatric Rating Scale [68], Structured Clinical Interview for the *DSM-5* (*Diagnostic and Statistical Manual of Mental Disorders* [Fifth Edition]) [69], Social and Occupational Functioning Assessment Scale (SOFAS) [70], and the Positive and Negative Syndrome Scale (PANSS) [71] will be used to assess change in general psychopathology and symptoms.

##### Psychological Treatment Target

The highest level in exposure hierarchies was achieved in each session. For each participant, the final target score of each of these measures will be the average over the 12 implementation phase sessions.

##### Neuropsychological Treatment Target

This will be operationalized as the difference (ie, change) between the first half and the second half of the VR exposure exercises in average power of high β band activity. A mean score will be used for analysis and will be given by the average change in ratings over the sessions attended.

### Planned Analyses

As this study is a pilot study designed to generate initial data to guide future research, a power calculation has not been conducted. A sample size of 10 participants is consistent with similar proof-of-concept and pilot studies [44,48].

### Primary Analyses

Means and SDs of acceptability, feasibility, safety, and usability measures and the percentage of participants showing different levels of ratings on the Pilot Study Assessment Instrument will be reported. Thematic analysis of the semistructured interviews will also be conducted. The number of adverse events and serious adverse events will be reported and reviewed.

### Secondary Analyses

#### Clinical Efficacy

Effect sizes, paired *t* tests and Wilcoxon analysis of clinical rating changes before and after the intervention will be computed, which will inform future efficacy trials.

#### Target Scores

Means, SDs, and 95% CIs of neural (average β band power) and psychological (exposure hierarchy level) target scores will be reported. The magnitude of the changes in both scores will reflect the degree of target engagement achieved. A mean score will be used for analysis and will be given by the average change over the first half (mean of weeks 3 to 8) and second half (mean of weeks 9 to 14) of the sessions attended.

#### Further Analyses

Whether greater engagement of neural and psychological targets is associated with greater change in clinical measures will also be explored. This finding would indicate a treatment mechanism that could be systematically tested in future studies.

## Results

As of February 2025, Hybrid has commenced (unblinded) recruitment activities from Orygen clinical services in Northwestern Melbourne, Australia. A total of 75 individuals have been approached and 64 individuals have been prescreened (41 individuals deemed eligible, 15 individuals ineligible, and 8 individuals declined or did not respond to contact attempts). Of the 41 individuals who were prescreened as eligible, 9 individuals consented (16 individuals declined and 8 individuals did not respond) and 5 individuals have been included in the study. Of the 5 individuals who have commenced the Hybrid treatment, 4 individuals are actively engaged in the program and 1 individual has withdrawn. The demographic information for these 5 participants are as follows: aged 21 to 27 (mean 24, SD 2) years; 5 male sex at birth consisting of gender identity of 2 cisgender males, 1 transgender male, 1 nonbinary, and 1 questioning their gender. We expect recruitment to conclude in July 2025 and for the results to be published in 2026.

## Discussion

### Anticipated Findings

Hybrid introduces a new treatment approach that integrates CBTp with VR and neurofeedback. This integration of treatment components may augment each individual element in such a way that it maximizes the impact of each individual component. We anticipate that this will produce durable skill-building and a “real world” transfer effect that motivates the use of Hybrid strategies in everyday life. In addition, individuals should see a reduction in AVH distress, severity, and frequency as evidenced by symptom change scores at the end of the treatment package.

We predict that Hybrid will be successful in achieving this based on the fact that the treatment package will be as follows. (1) Engaging and acceptable: previous studies have shown that young people in the early stages of psychosis find neurophysiological assessments highly acceptable and the neurofeedback and VR components of Hybrid leverage “gamification” [72,73] (ie, using game design and mechanics to encourage active participation) in the treatment of mental disorders. (2) Easily implementable given the increasingly affordable and portable nature of the tools used in the Hybrid intervention (EEG and VR). (3) Empowering: young people develop self-directed control over their symptoms, as opposed to being an “object” of treatment, which is often how other treatment approaches, such as pharmacotherapy, are experienced [74]. As has been recognized [75] previously, neurofeedback can be a means of “taking back control” of the brain by promoting strategies to manage symptoms that come “from the inside” and can be used at any point in the course of everyday life. (4) Focused on individual symptoms rather than diagnostic categories. This is in keeping with the current trend in psychiatric research away from disorder-bound approaches toward transdiagnostic and symptom-based risk factors [76,77]. Therefore, Hybrid could represent a breakthrough in clinical research for effective treatments for psychosis and, if successful, would significantly improve clinical outcomes in young people with AVH*.*

In terms of study limitations, we highlight that our use of the PANSS and SOFAS as measures of symptoms and functioning may be suboptimal for fine-grained assessment of symptomatology (such as for the negative symptoms) and function. We note that the main aim of this study is to assess the feasibility and acceptability of the Hybrid treatment and we plan to include additional, more fine-grained measures in future studies. We chose to use the PANSS and SOFAS based on many previous studies in early psychosis populations using these measures and increasing our comparability to previous research [70,71]. Second, our clinical assessments are structured to minimize the burden on the individual, therefore, certain features which may be of significance (eg, past history of trauma or eating disorders) were not assessed, but may affect responsiveness to the Hybrid treatment. In subsequent trials, a more thorough assessment of psychopathology may be included to address this. Finally, by virtue of design, we cannot make inferences about any other symptoms associated with psychosis (eg, paranoia and disorganized thinking) as we were targeting AVH in these efficacy trials. Future studies could expand to target other psychotic symptoms.

### Conclusion

This pilot study aims to demonstrate the safety, feasibility, and acceptability, as well as establish proof of concept for Hybrid treatment. This initial study will provide valuable information as to whether the Hybrid treatment protocol could be modified and whether moving on to efficacy trials is indicated.

## Abbreviations

AVH: auditory verbal hallucination
CBT: cognitive behavioral therapy
CBTp: cognitive behavioral therapy for psychosis
DSM-5: Diagnostic and Statistical Manual of Mental Disorders (Fifth Edition)
EEG: electroencephalography
ERP: exposure and response prevention
FEP: first episode psychosis
OCD: obsessive-compulsive disorder
PANSS: Positive and Negative Syndrome Scale
PTSD: posttraumatic stress disorder
rtfMRI: real-time functional magnetic resonance imaging
SOFAS: Social and Occupational Functioning Assessment Scale
VR: virtual reality

## Data Availability

The datasets generated or analyzed during this study are available from the corresponding author on reasonable request.
